# Use of Non-Linear Ultrasonic Techniques to Detect Cracks Due to Steel Corrosion in Reinforced Concrete Structures

**DOI:** 10.3390/ma12050813

**Published:** 2019-03-09

**Authors:** Miguel Ángel Climent, Marina Miró, Jesús Carbajo, Pedro Poveda, Guillem de Vera, Jaime Ramis

**Affiliations:** 1 Civil Engineering Department, University of Alicante, 03690 Sant Vicent del Raspeig, Alicante, Spain; m.miro@ua.es (M.M.); guillem.vera@ua.es (G.d.V.); 2 DFISTS, University of Alicante, 03690 Sant Vicent del Raspeig, Alicante, Spain; jesus.carbajo@ua.es (J.C.); pedro.poveda@ua.es (P.P.); jramis@ua.es (J.R.)

**Keywords:** corrosion, steel, reinforced concrete, cracking, ultrasonic wave, non-destructive detection

## Abstract

In this work, non-linear ultrasonic wave techniques have been used to detect the onset of micro-cracking due to steel corrosion in model reinforced concrete elements. The specimens were of prismatic shape with a single steel rebar. The corrosion was forced by admixing an appropriate amount of sodium chloride at the moment of preparing the concrete mix, and by the application of an electric field, using a constant current density power source, and making the steel rebar work as the anode, and an external counter-electrode as the cathode. The preliminary results indicate that the onset of cracking seems to be accompanied by the appearance of higher-harmonic generation at the output signal (harmonic distortion), when the system is excited by the means of an ultrasound wave with a burst central frequency. Other phenomena related to the micro-cracks induced by corrosion, such is the parametric generation with respect to the fundamental amplitude, have not been observed until now.

## 1. Introduction

The corrosion of steel is one of the main mechanisms limiting the service life of reinforced and pre-stressed concrete structures, both in buildings and civil infrastructure [1]. The expansive character of the steel corrosion products is at the origin of the damage suffered by concrete [2], whose early symptoms are micro- and macro-cracking [3,4], followed by the spalling or the delamination of the concrete cover. Further consequences of the corrosion of embedded steel are the following: loss of bonding between concrete and steel, loss of steel ductility, and loss of the cross-sectional area of steel [5]. These phenomena contribute to the reduction of the serviceability and load-bearing capacity of the structures. Usually, the appearance of cracking is considered to be the limit state, with regard to the durability of concrete structures that are affected by steel reinforcement corrosion [6,7].

There is much interest in developing non-destructive appraisal techniques that are able to provide early warnings for corrosion initiation. These techniques would be very useful for the surveying and inspection of structures in the field of maintenance and repair of constructions affected by reinforcement corrosion. The so-called electrochemical techniques have been widely used for assessing the state and risk of corrosion of steel in concrete [8,9,10,11]. The measurement of the steel corrosion potential (E_corr_) provides qualitative information on the corrosion state of steel, which is helpful for mapping the corrosion risk for a structural member. The corrosion rate indicates the steel corrosion activity, which is usually expressed in electrochemical units (µA/cm^2^). This latter parameter can even be used in mathematical models for predicting the remaining service life of a structure that is affected by steel reinforcement corrosion [7]. However, electrochemical techniques do not provide information about micro-cracking that is induced in concrete by the generation of the corrosion products of steel.

Several techniques, based on the use of elastic waves, have been used for assessing mechanical properties, and for the detection of damage in materials and structures. The waves propagating through a material can react to defects, voids, and discontinuities such as cracks [12]. The ultrasonic pulse velocity (UPV) method has been widely used for the inspection of concrete structures. The UPV technique, based on the measurement of the speed of sound, is considered to be sensitive to the state of the interface between concrete and steel, and also to cracking [13]. Nevertheless, the UPV measurements through concrete are less sensitive to the distributed micro-cracks appearing during the early stages of corrosion of steel in concrete [14]. The impact echo (IE) technique, which is based on applying a mechanical impact, and then monitoring the signals that are received by sensors located on the concrete surface, has been scarcely used for detecting the damage induced by corrosion of embedded steel [15]. Some authors have indicated that the reliability of the IE method decreases with an increase of the thickness of the concrete members [12]. The acoustic emission (AE) technique has been commonly used for detecting active deformation processes: crack growth, void closures, plastic deformations, etc. [12,16]. There are specific recommendations for the application of the AE method toward the evaluation of damage in concrete [17]. However, an important limitation of the AE technique is that it is not able to detect passive defects [12], thus reducing the applicability of AE to occasional surveys of existing reinforced concrete structures. 

Non-linear ultrasonic (NLU) techniques have been shown to be useful for the evaluation of material degradation [18]. Specifically, they are helpful for detecting cracking at early stages [19]. The NLU technique is based on the fact that the non-linear interaction of ultrasound with cracked materials generates higher acoustic harmonics [14,19,20,21,22,23]. 

Many experimental techniques exist for detecting the micro-cracking of concrete [24,25]. Special mention should be given to methods based on digital image analysis, which have been used for investigating damage, cracks, and fracture mechanics parameters in concrete and other construction materials [26,27]. However, the aim of the present work is to study the possibility of using the NLU technique for the non-destructive evaluation of damage in concrete elements, due to the corrosion of embedded steel. To this end, NLU measurements have been performed on model-reinforced concrete specimens that are subjected to forced steel corrosion, due to the application of an electric field. The preliminary results suggest that it is possible to detect the micro-cracks that are generated by the expansion of steel corrosion products by using the NLU technique. The technique can be considered as a non-destructive methodology that is complementary to the classical electrochemical techniques, since these latter are not sensitive to the appearance of the abovementioned micro-cracking.

## 2. Materials and Methods 

Testing was conducted on reinforced cement mortar elements. The specimens were of prismatic shape, with a single deformed steel rebar as the reinforcement. The choice of using cement mortar instead of concrete was due to the interest in using a more homogeneous and simple model material, by avoiding the presence of coarse aggregate, which could create heterogeneities at the steel-concrete interface. The corrosion of steel was forced by the application of an electric field, between the steel rebar (anode), and an external cathode. NLU measurements were taken during the corrosion test. The appearance of cracking at the concrete surface was detected by using a microscope, which also allowed for the measurement of the crack width. All of experimental details are described below. 

### 3.1. Preparation of the Reinforced Cement Mortar Specimens

**Figure 1:**
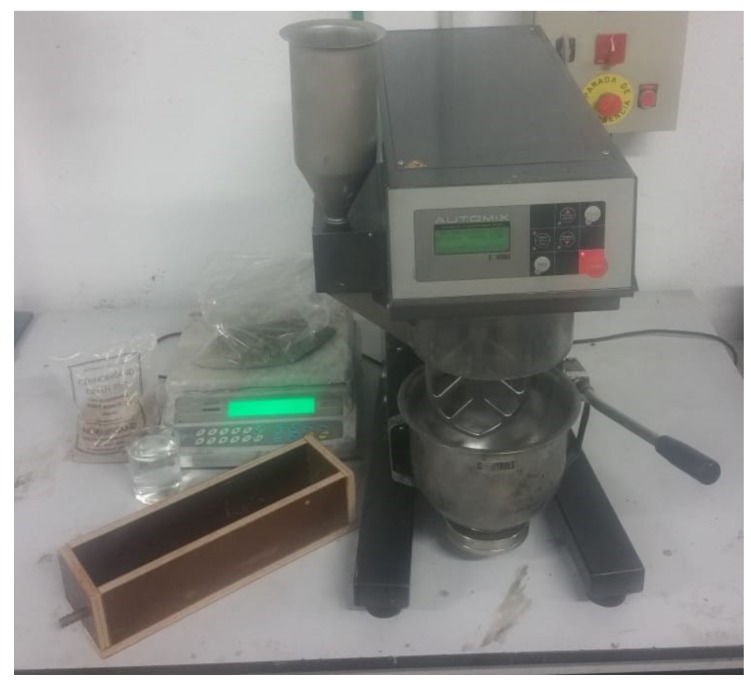
Photo of the wooden mold with the steel bar, the mortar mixer, and the materials used: cement (on top of the balance), siliceous sand, and water containing the proper amount of dissolved NaCl.

The cement mortar was prepared with a sulfate-resisting ordinary Portland cement, CEM I 52,5 R SR(3), following the European standard [28]. The aggregate was standard siliceous sand. The water–cement ratio (w/c) of the mortar was 0.5, and finally sodium chloride was dissolved into the mixing water to obtain a content of 2% Cl^-^ relative to the cement weight in the hardened mortar. The composition details are given in Table 1. Figure 1 shows a photo with the wooden mold, the mortar mixer, and the materials used in the mix.

**Figure 2:**
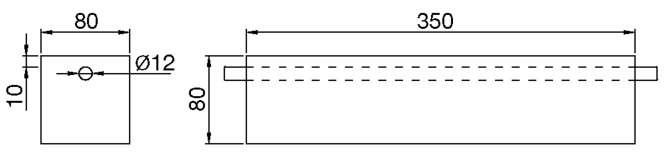
Scheme of the reinforced cement mortar specimens. All dimensions in mm.

The mortar specimens were of prismatic shape, with dimensions 8 × 8 × 35 cm^3^. They were cast in wooden molds, containing a single deformed steel bar (diameter Ø 12 mm), located in one of the main symmetry planes of the specimen, leaving a concrete cover depth of 10 mm (see Figure 1 and Figure 2). Before putting the steel bar into the empty mold, the steel surface was cleaned from the native corrosion products, following a recommended procedure [29], and the bar was weighed. The ends of the steel bar were protected with vinyl electric tape, to avoid the triple contact steel-mortar-air, leaving an exposed steel area of 120 cm^2^ (the steel-mortar surface of contact). The mortar specimens were compacted mechanically, finished, and left in the molds over 24 h. After demolding, the specimens were cured over seven days in a humid chamber (20 °C and 95% relative humidity). During the stage in the humid chamber, and before starting the forced corrosion test, the spontaneous corrosion rate of the steel bar was measured through the linear polarization technique, using the portable corrosion rate meter Gecor8 (Geocisa, Madrid, Spain).

### 3.2. Corrosion Tests

**Figure 3:**
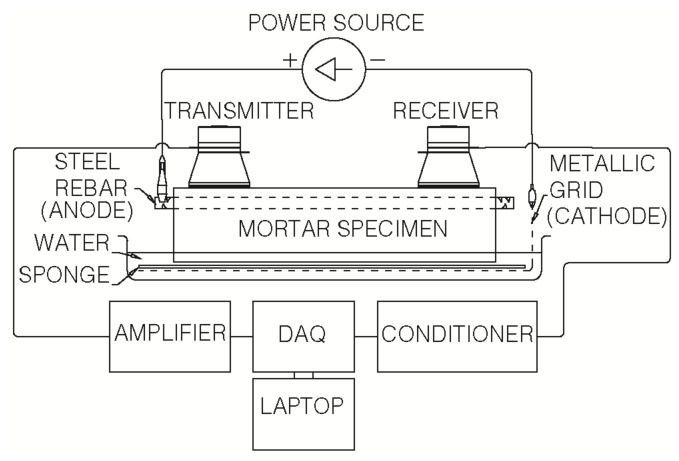
Schematic representation of the setup of the forced corrosion tests, also showing the system that is used for performing non-linear ultrasonic measurements.

The forced corrosion tests were performed by applying an electric field between the steel rebar (anode) and an external cathode, consisting of a galvanized steel grid. The tests, which lasted 23 days, were run in galvanostatic conditions (a constant anodic current density of 100 µA/cm^2^), using an electrophoretic power source. During the tests, the bottom of the mortar specimens were kept in permanent contact with tap water (the maximum height of the contact between water and mortar is 5 mm), in order to maintain an adequate level of electric conductivity for the material. The mortar specimen was put on top of the cathode, with a polypropylene sponge in between them (see Figure 3).

At the end of the corrosion tests, the mortar specimens were broken, and the corroded steel rebar was extracted. The bar was cleaned to eliminate the corrosion products [29], and it was weighed again, in order to determine the steel mass loss at the end of the corrosion test. The gravimetrically determined mass loss is designated as ∆m_G_. This mass loss value was compared to the theoretically calculated mass loss, obtained by the application of the Faraday law with the relevant parameter values of the test [3]. The theoretical (or electrochemical) mass loss is designated as ∆m_T_.

Due to the chosen setup and geometric conditions of the experiments, the cracks due to steel corrosion appeared at the upper surface of the mortar specimens, which allowed an easy periodic inspection of this surface with a crack width microscope (magnification 40×, model 58-C0218, Controls, Milan, Italy). In this way, it was possible to detect the appearance of the first surface micro-crack, and the posterior monitoring of the growth of the crack width with time.

The number of reinforced cement mortar specimens tested in this work was three, which were designated as M1, M2, and M3.

### 3.3. Non-Linear Ultrasonic Measurements

**Figure 4:**
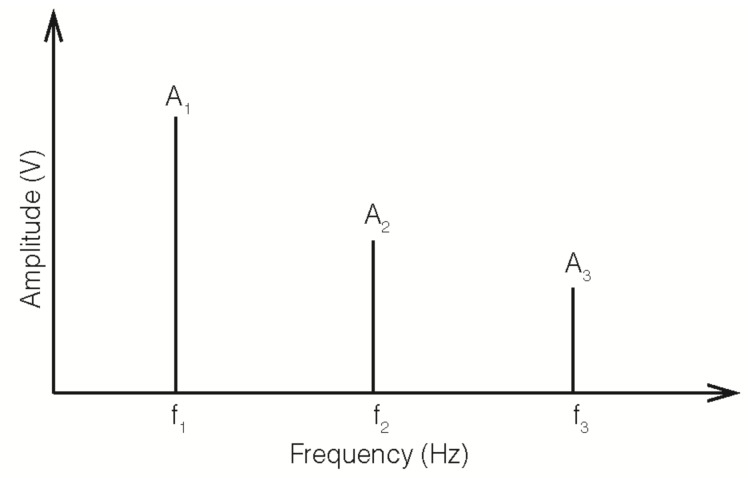
Frequency spectrum showing the emitted frequency (f_1_), and two of the generated harmonics (f_2_ and f_3_).

A schematic representation of the experimental setup used for the NLU measurements is shown in Figure 3. It consists of two piezoelectric ceramic ultrasonic transducers (UCE-UT-28100 PZT-8, Ucesonic, Beijing, China), a signal conditioner type 2698-A-0F4 (Brüel&Kjaer, Naerum, Denmark), an audio amplifier interM M-500 (South Korea), and a DAQ (data acquisition system, National Instruments, Austin, TX, USA) NI USB 6361 connected to a laptop (Lenovo Ideapad, Quarry Bay, Hong Kong, China). It is necessary to emphasize that NLU measurements were obtained through a contact-based technique. Both ultrasonic transducers are glued at each end of the upper face of the mortar specimen, one of them working as a transmitter, and the other as a receiver. The NLU technique involves the generation of a fundamental sinusoidal signal A_1_, which is amplified and sent to the transmitter. In this study, the fundamental signal frequency f_1_ was selected at a value of 30 kHz. After propagation through the specimen, the ultrasonic wave reached the other transducer; the received signal was conditioned and post-processed so as to obtain its frequency spectrum (see Figure 4). This spectrum lets us obtain the variation of the amplitudes of the higher order harmonics (in our case, the second, A_2_, at a frequency f_2_ = 60 kHz, and the third, A_3_, at a frequency f_3_ = 90 kHz) to that of the emitted frequency (the fundamental, A_1_), and thus obtain the parameters of nonlinearity [18,19,21], which are herein defined as:

β = A_2_/A_1_^2^ (1)

β’ = A_3_/A_1_^3^ (2)

Given that the nonlinear parameters are sensitive to the microscale variation of the propagating medium, these may not only serve as an indicator of the micro-cracking phenomena occurring, due to the forced corrosion, but also for the early-stage damage evaluation in reinforced concrete. In order to assess the variation thereof, the described procedure was repeated continuously by the means of an automated routine every 10 minutes, throughout the entire NLU test (i.e., around 4000 times per test). Once the variation ratio of β or β’ relative to the initial value was found to be higher than 10, the elapsed time was recorded and taken, as the time that is needed for the generation of micro-cracking, due to the corrosion process. It should be noted that the ratio value that is used in these calculations was chosen, based on the trend of the nonlinear parameters for the tests carried out.

## 3. Results and Discussion

The surface cracking of reinforced concrete due to embedded steel corrosion in the presence of chloride ions, has been studied previously by a number of authors [3,4,30,31,32]. The cracking process is usually considered as consisting of two steps. The first part, called the generation step, is the time that has elapsed since the onset of the corrosion process (the depassivation of steel), and the appearance of the first visible crack at the surface of the concrete [3]. The second part, called the propagation step, is the period during which the crack width keeps growing [3]. 

It is known that for an experimental setup similar to that used in this work, a small amount of loss of effective radius of the rebar of about 15 µm to 35 µm is enough to produce the first appearance of a visible crack at the concrete surface (a crack width that is equal to or less than 0.05 mm) [3]. Regarding the propagation step, it is considered that the crack width increases linearly with the propagation of corrosion; i.e., it increases linearly with the loss of effective radius of the rebar [3]. It is assumed that uniform corrosion products are formed around the steel surface to simplify the analysis, although corrosion takes place in chloride-contaminated concrete as pitting corrosion; therefore it is non-uniform. However, as pitting corrosion progresses, it appears as uniform corrosion, and the uniform corrosion assumption can be considered to be reasonable [4]. Following this type of analysis, in this work, we have calculated the progress of the corrosion process; i.e., the theoretical loss of effective radius of the rebar, by applying the Faraday law with the relevant parameters of our tests, considering the corrosion process as a generalized and homogeneously distributed process [30,31,32]. For a constant anodic current density (I_corr_), the loss of the effective radius of the steel rebar (x) can be calculated as [31]:

x = 0.0319 × I_corr_ × t (3)

where x is expressed in µm, I_corr_ is expressed in µA/cm^2^, and t is the time elapsed since the beginning of the forced corrosion test, in days. The value 0.0319 contains all of the relevant physical constants and unit change factors that are needed for the calculation. In other works, x is termed as the penetration of corrosion, and designated by the symbol P_x_ [32].

**Figure 5:**
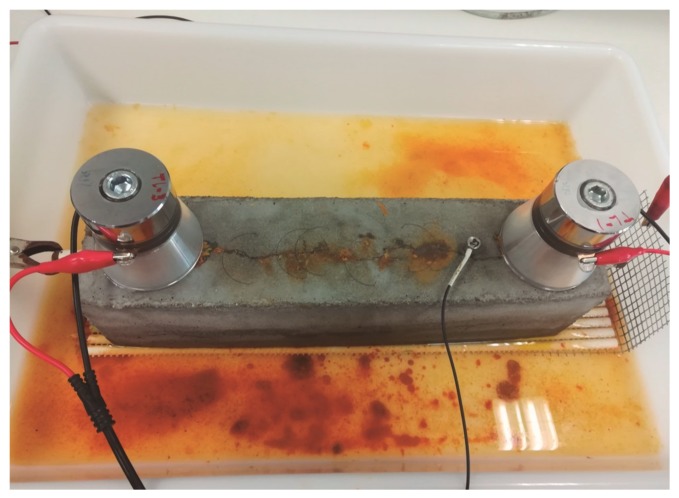
Photo of the M3-reinforced mortar specimen near the end of the corrosion test.

Figure 5 shows a photo of the M3-reinforced mortar specimen near the end of the forced corrosion test. An appreciable pattern of cracking occurred at the upper surface of the specimen. This cracking was longitudinal to the steel rebar, and some liquid-containing products of the corrosion of steel leaked out of the mortar specimen through the open cracks. The pH of this liquid was measured, obtaining a value of 3, in good agreement with the previous findings of other authors [30].

**Figure 6:**
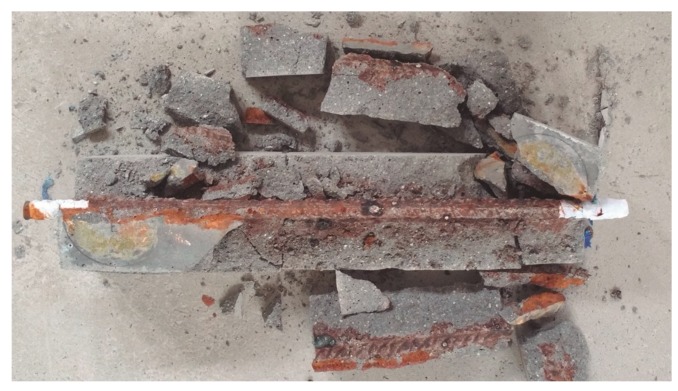
Photo taken after breaking the M1-reinforced mortar specimen at the end of the corrosion test.

At the end of the forced corrosion test, the mortar specimens were broken, and the steel bar was extracted. Figure 6 shows a photo that was taken just after breaking the M1 specimen. It is appreciable that the corrosion process had affected the whole steel bar. However, some spots of more intense corrosion (pits), were also observed. These pits were more clearly appreciable after cleaning the corroded bar to remove the corrosion products; see Experimental Procedures. The appearance of the steel bars after the corrosion tests of the other specimens was similar to that shown in Figure 6.

Since the mix that was used for the reinforced cement mortar specimens contained 2% Cl^−^ relative to the cement weight, it was important to quantify the spontaneous corrosion that was suffered by steel before the forced corrosion test, i.e., during the curing period, and the time elapsed before starting the forced corrosion test. This was done by measuring the spontaneous corrosion rate by using a portable corrosion rate meter, see Experimental Procedure, with a typical frequency of one measurement every two days. The recorded corrosion rate values were always in the range from 0.7 µA/cm^2^ to 3 µA/cm^2^. Since the impressed anodic current density during the forced corrosion test was 100 µA/cm^2^, the spontaneous corrosion prior to the forced corrosion test, was considered as negligible, thus allowing for the use of Equation (3) for the estimation of the progress of the corrosion process. Nevertheless, a further check of the validity of this assumption was obtained by measuring the experimental mass loss of the steel rebar at the end of the corrosion test (∆m_G_), comparing it with the theoretical weight loss, or electrochemical weight loss (∆m_T_), calculated according to the application of the Faraday law with the relevant parameters of the tests. See the Experimental Procedure. For an anodic current density of 100 µA/cm^2^, an exposed steel surface of 120 cm^2^, and a duration of the forced corrosion test of 23 days, the theoretical weight loss was 6.90 g.

Table 2 shows the comparison between the experimentally determined mass losses and the theoretical mass loss of the steel bars in these tests. The results indicate a good concordance between the experimental and theoretical values, which confirms the validity of assuming that the corrosion of steel before the forced corrosion test is negligible. Another consideration that can be derived from the data of Table 2 is that the accelerated (forced) corrosion processes have been developed, with a current efficiency that is close to 100%, which is typical of cases of accelerated corrosion tests that are performed on specimens of reinforced concrete that are contaminated with chlorides and with no limitation of water in the system [33].

**Figure 7:**
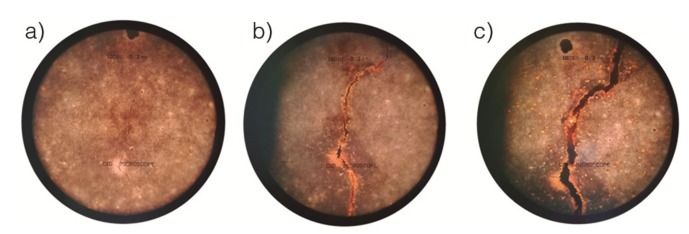
Microscopic observation of the evolution of a crack corresponding to one of the tested specimens. (**a**): Three days of passing current; (**b**): 11 days; (**c**): 23 days.

A microscope was used for detecting the appearance of cracks at the mortar surface. This also allowed for the monitoring of the evolution of the crack widths with time. Figure 7 shows three photos that correspond to the evolution of one of the visible cracks at the surface of one of the tested specimens.

**Figure 8:**
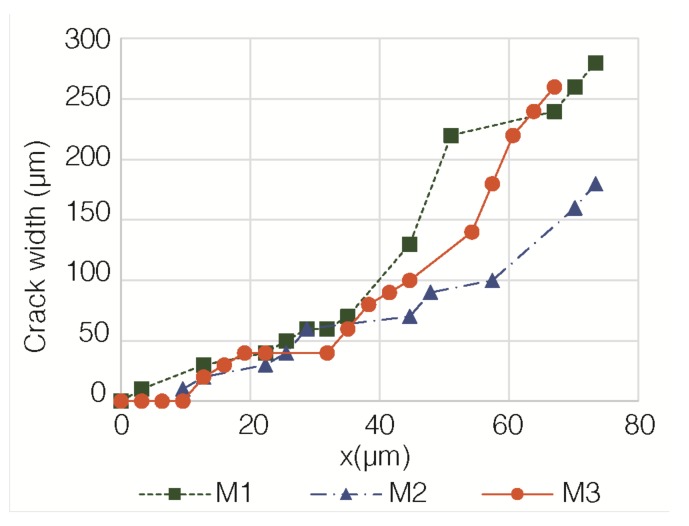
Evolution of the crack widths of the mortar specimens in function of the losses of the effective radius of the steel bars, calculated through Equation (3).

Figure 8 depicts the evolution of the crack widths of the specimens M1, M2, and M3, in function of the progress of the corrosion process, i.e., in function of the losses of effective radius of the steel rebar, calculated through Equation (3). It can be appreciated that the observed evolution is very similar for the three tested specimens. A first stage of the corrosion process seems to exist, up to approximately 10 µm of radius loss (for the cases studied here), during which there is no visible crack. This would be the generation step following Andrade and co-workers [3,30,31,32]. Afterwards, the crack width shows an approximately linear increase with the radius loss. This would be the generation step [3,30,31,32]. Table 3 shows the parameters of the least-squares linear fitting of the crack width (w) with x, corresponding to the data of Figure 8. The fitting was done according to the following equation:

w = a + b × x(4)

where w is the crack width (µm) and x is the radius loss of the steel bar, due to corrosion (µm). The results that are shown in Table 3 confirm the approximately linear increase of w with x during the generation step, in agreement with the findings of previous authors [3].

One of the key points in this research was the detection of the cracking of the mortar specimens due to corrosion of the steel rebar. This has been achieved in this work by the direct microscopic observation of the upper surface of the specimens, see Figure 7. Besides, NLU measurements were taken periodically during the forced corrosion tests, in order to ascertain the capability of the NLU technique for the early monitoring of the cracking, due to the embedded steel corrosion. The feature event of the NLU measurements, indicating the appearance of micro-cracking, is the generation of higher acoustic harmonics [14,19,20,21,22,23]. The quantitative criterion that is adopted in this work is the variation ratio of the nonlinearity parameters (β or β’), relative to the initial value, which is higher than 10 (see Experimental Procedure).

**Figure 9:**
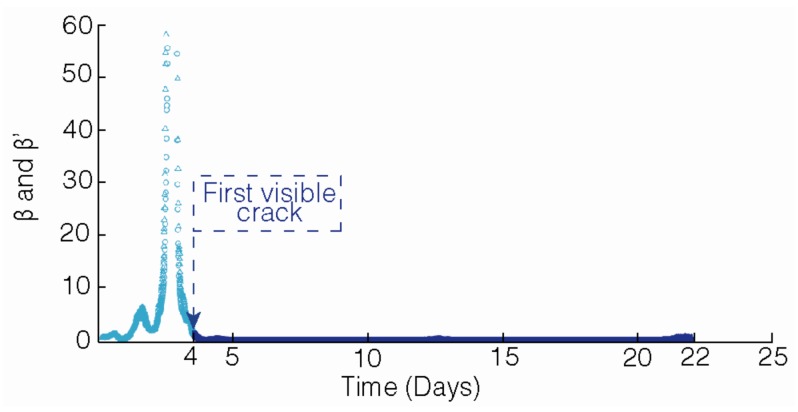
Evolution of the nonlinearity parameters (derived from the non-linear ultrasonic (NLU) measurements) along the duration of the forced corrosion test of specimen M3. β: ○; β’: Δ.

Figure 9 depicts the variation with time of the nonlinearity parameters that were derived from NLU measurements during the forced corrosion test of specimen M3. The abscissa axis of Figure 9 represents the time elapsed since the starting of passing current. It is appreciable from Figure 9 that both β and β’ exceeded the established threshold of the 10-fold increase relative to the initial values, approximately two-and-a-half days after the beginning of the forced corrosion test. In this case, the first microscopic observation of cracking at the upper surface of the M3 specimen was at the fourth day after starting the test. Hence, these preliminary results seem to indicate that the microscopic observation of cracking of reinforced mortar due to steel corrosion was accompanied by a clear increase of the nonlinearity parameters. This finding is in agreement with the previous observations of other authors [23,34]. For the rest of the tested mortar specimens, the behavior that was found is similar to that which is shown in Figure 9, although the corresponding figures are not shown here for the sake of brevity of the manuscript.

Table 4 shows the relevant parameters that are related to the detection of cracking of the reinforced mortar specimens, due to steel rebar corrosion. Both for the direct microscopic observation and for the indirect detection, based on the variation of the nonlinearity parameters derived from the NLU measurements, Table 4 shows the time that is elapsed for when the cracking is detected, and the corresponding losses of the effective radius of the rebar that are calculated through Equation (3). It is appreciable from the table that the detection times are very similar, although it is not possible now to establish whether the NLU measurements can detect the cracking earlier than direct microscopic observations. Nevertheless, it must be considered that using an automated system for the NLU measurements would represent a much lower experimental effort that is needed for detecting the onset of cracking, than that through using direct microscopic observation. Regarding the amount of steel corrosion that is needed for cracking mortar or concrete, a comparison of the values of x_1_ (microscopic observation) and x_2_ (NLU measurements) in Table 4 indicate that both detection systems lead to similar values of the penetration of corrosion that is needed for cracking the mortar. These values range between 6 µm and 13 µm (losses of steel bar radius) for the tests of this work. Furthermore, these values are in good agreement with the previous findings of other authors who established that a small penetration of corrosion is enough for generating the first visible crack, due to corrosion in reinforced concrete [3,4]. An empirical relationship was proposed for describing the relationship between the minimum amount of corrosion that is needed for cracking (x_0_), and the quotient c/Ø [3]:

X_0_ = 7.53 + 9.32 c/Ø (5)

where x_0_ is expressed in µm, c stands for the concrete cover depth over the steel bar (mm), and Ø stands for the bar diameter (mm). Applying Equation (5) with the relevant geometric parameters of the reinforced mortar specimens tested in this work, a value of x_0_ of 15.3 µm is derived, which is in fairly good agreement with the x values that are reported in Table 4. 

The observations in this work seem to indicate that it is possible to use the NLU technique for the detection of the cracking of reinforced concrete, due to the corrosion of the embedded steel. However, this can be considered only as a preliminary result. Further research is necessary to ascertain whether the NLU technique can be extensively used, both for research purposes, and for practice engineering surveys of reinforced concrete structures. In the latter case, differences may be found, regarding, for instance, the steel corrosion rate, which is usually much lower in the case of real-life structures, compared to the values used in this work. Another difficulty which can be found is related to the accessibility of concrete outer surfaces for locating the ultrasonic transducers that need to be close to the corroding steel bars. Particularly, the technique should be tested on bigger and more complex reinforced concrete specimens, and on construction elements such as concrete beams, columns, footings, etc. It is hoped those future studies will provide an optimized protocol indicating where, when to apply the technique, and how many measurements are needed for a proper detection of the cracking of corroding reinforced concrete structures. 

## 4. Conclusions

The preliminary results that are obtained in this work indicate that it is possible to use non-linear ultrasonic techniques for the detection of cracking of model-reinforced cement mortar specimens, due to the corrosion of the embedded steel rebar. It is apparent that the onset of micro-cracking is accompanied by the appearance of higher harmonic generation at the output signal, when the system is excited by the means of an ultrasound with a burst central frequency. It seems that the event of micro-cracking can be associated with an increase in the nonlinearity parameters to values of at least 10 times higher than the initial values. The use of non-linear ultrasonic measurements yield similar values of times for cracking (or the amount of corrosion that is necessary for cracking), as compared to the direct visual or microscopic observations of the mortar surface. This would reduce the experimental effort that is needed in research, that is aimed at detecting the onset of cracking, especially if automated NLU measurements are used. Nevertheless, the necessary limitation of the extension of this preliminary work does not allow generalizations of this conclusion. More research is necessary to ascertain whether the NLU measurements can be extensively used, both for research purposes, and for pilot engineering surveys of corroding reinforced or pre-stressed concrete structures.

## Figures and Tables

**Table 1:** Composition of the cement mortar.

Material	Amount (g)
Cement (CEM I 52,5 R SR(3))	450
Standard siliceous sand	1350
Deionized water	225 (w/c = 0.5)
NaCl	14.8 (2% Cl^−^ relative to cement weight)

**Table 2:** Comparison of the gravimetric and theoretical mass losses of the steel bars tested.

Specimen	∆m_G_ (g)	∆m_G_/∆m_T_
M1	6.07	0.880
M2	8.64	1.252
M3	6.92	1.003

**Table 3:** Fitting parameters of Equation (4) to the sets of data of Figure 8.

Specimen	a	b	r^2^ (Correlation Coefficient)	Intercept with the Abscissa Axis (µm)
M1	−32.811	4.029	0.9276	8.1
M2	−19.990	2.447	0.9473	8.2
M3	−34.587	3.705	0.8992	9.3

**Table 4:** The relevant parameters of the detection of the cracking of the tested specimens.

Specimen	Microscope		NLU measurements	
	t_1_ (days)	x_1_ (µm)	t_2_ (days)	x_2_ (µm)
M1	2	6.4	3*	9.6
M2	4	12.8	4.01	12.8
M3	4	12.8	2.47	7.9

* The NLU measurements for M1 were performed without using the automated system, see the Experimental Procedure.

## References

[1] ACI Committee 222R-96 (1996). Corrosion of Metals in Concrete.

[2] Suda K., Misra S., Motohashi K. (1993). Corrosion products of reinforcing bars embedded in concrete. Corros. Sci..

[3] Alonso C., Andrade C., Rodríguez J., Díez J.M. (1998). Factors controlling cracking of concrete affected by reinforcement corrosion. Mater. Struct..

[4] Liu Y., Weyers R.E. (1998). Modeling the time-to-corrosion cracking in chloride contaminated reinforced concrete structures. ACI Mater. J..

[5] Bertolini L., Elsener B., Pedeferri P., Polder R. (2004). Corrosion of Steel in Concrete.

[6] Uomoto T., Misra S. (1988). Behavior of concrete beams and columns in marine environment when corrosion of reinforcing bars takes place. Proceedings of the 2nd International Conference on Concrete in Marine Environment.

[7] (2010). EHE-08, Instrucción de Hormigón Estructural (EHE-08, Spanish Structural Concrete Code).

[8] (1991). ASTM C 876–91, Standard Test Method for Half-Cell Potential of Reinforcing Steel in Concrete.

[9] Elsener B., Andrade C., Gulikers J., Polder R., Raupach M. (2003). Recommendation on half-cell potential measurements. Mater. Struct..

[10] Andrade C., González J.A. (1978). Quantitative measurements of corrosion rate of reinforcing steels embedded in concrete using polarization resistance measurements. Werkst. Korros..

[11] Andrade C., Alonso C., Fullea J., Polimón J., Rodriguez J. (2000). Proceedings of the International Workshop on Measurement and Interpretation of the On-site Corrosion Rate (MESINA), Madrid, Spain, 22–23 February 1999.

[12] Zaki A., Chai H.K., Aggelis D.G., Alver N. (2015). Non-destructive evaluation for corrosion monitoring in concrete: A review and capability of acoustic emission technique. Sensors.

[13] Sharma S., Mukherjee A. (2010). Longitudinal guided waves for monitoring chloride corrosion in reinforcing bars in concrete. Struct. Health Monit..

[14] Shah A.A., Ribakov Y. (2009). Non-destructive evaluation of concrete in damaged and undamaged states. Mater. Des..

[15] Liang M.T., Su P.J. (2001). Detection of the corrosion damage of rebar in concrete using impact-echo method. Cem. Concr. Res..

[16] Calabrese I., Campanella G., Proverbio E. (2013). Identification of corrosion mechanisms by univariate and multivariate statistical analysis during long term acoustic emission monitoring on a pre-stressed concrete beam. Corros. Sci..

[17] Ohtsu M. (2010). Recommendation of RILEM TC 212-ACD: Acoustic emission and related NDE techniques for crack detection and damage evaluation in concrete. Mater. Struct..

[18] Jhang K.Y. (2009). Nonlinear ultrasonic techniques for non-destructive assessment of microdamage in materials: A review. Int. J. Precis. Eng. Manuf..

[19] Shah A.A., Ribakov Y., Hirose S. (2009). Nondestructive evaluation of damaged concrete using nonlinear ultrasonics. Mater. Des..

[20] Xiang H., Newtson C.M., Woodward C. (2008). Optimization of nonlinear ultrasound results to determine dynamic properties of concrete. J. Mater. Civ. Eng..

[21] Shah A.A., Ribakov Y. (2009). Non-linear ultrasonic evaluation of damaged concrete based on higher order harmonic generation. Mater. Des..

[22] Shah A.A., Ribakov Y., Zhang Ch. (2013). Efficiency and sensitivity of linear and non-linear ultrasonics to identifying micro and macro-scale defects in concrete. Mater. Des..

[23] Korenska M., Matysik M., Vyroubal P., Pospisil K. Assessment of reinforcement corrosion using nonlinear ultrasonic spectroscopy. Proceedings of the 5th NDT Progress—International Workshop of NDT Experts.

[24] Maji A.K. (1995). Review of noninvasive techniques for detecting microfracture. Adv. Cem. Based Mater..

[25] Golewski G.L. (2018). Evaluation of morphology and size of cracks of the interfacial transition zone (ITZ) in concrete containing fly ash (FA). J. Hazard.Mater..

[26] Ringot E., Bascoul A. (2001). About the analysis of microcracking in concrete. Cem. Concr. Compos..

[27] Golewski G.L., Sadowski T. (2016). Macroscopic evaluation of fracture processes in fly ash concrete. Solid State Phenom..

[28] UNE-EN 197-1 (2011) (2000). “Cemento. Parte 1: Composición, Especificaciones y Criterios de Conformidad de los Cementos Comunes” (“Cement. Part 1: Composition, Specifications and Conformity Criteria for Common Cements”).

[29] ASTM G 1-90 (1990). Standard Practice for Preparing, Cleaning and Evaluating Corrosion Test Specimens.

[30] Andrade C., Alonso C., Molina F.J. (1993). Cover cracking as a function of bar corrosion: Part I—Experimental test. Mater. Struct..

[31] Molina F.J., Alonso C., Andrade C. (1993). Cover cracking as a function of bar corrosion: Part II—Numerical model. Mater. Struct..

[32] Pedrosa F., Andrade C. (2017). Corrosion induced cracking: Effect of different corrosion rates on crack width evolution. Constr. Build. Mater..

[33] Nossoni G., Harichandran R. (2012). Current efficiency in accelerated corrosion testing of concrete. Corrosion (NACE).

[34] Woodward C., Amin M.N. (2008). Evaluating rebar corrosion using nonlinear ultrasound. AIP Conference Proceedings.

